# The GPR40 Agonist GW9508 Enhances Neutrophil Function to Aid Bacterial Clearance During *E. coli* Infections

**DOI:** 10.3389/fimmu.2020.573019

**Published:** 2020-09-29

**Authors:** Patricia R. Souza, Mary E. Walker, Nicolas J. Goulding, Jesmond Dalli, Mauro Perretti, Lucy V. Norling

**Affiliations:** ^1^The William Harvey Research Institute, Barts and The London School of Medicine and Dentistry, Queen Mary University of London, London, United Kingdom; ^2^Centre for Inflammation and Therapeutic Innovation, Queen Mary University of London, London, United Kingdom

**Keywords:** GPR40, neutrophil, resolvins, bacteria, resolution, lipoxins

## Abstract

G-protein-coupled receptor 40 (GPR40) is known to play a role in the regulation of fatty acids, insulin secretion, and inflammation. However, the function of this receptor in human neutrophils, one of the first leukocytes to arrive at the site of infection, remains to be fully elucidated. In the present study, we demonstrate that GPR40 is upregulated on activated human neutrophils and investigated the functional effects upon treatment with a selective agonist; GW9508. Interestingly, GPR40 expression was up-regulated after neutrophil stimulation with platelet-activating factor (10 nM) or leukotriene B_4_ (LTB_4_, 10 nM) suggesting potential regulatory roles for this receptor during inflammation. Indeed, GW9508 (1 and 10 μM) increased neutrophil chemotaxis in response to the chemokine IL-8 (30 ng/ml) and enhanced phagocytosis of *Escherichia coli* by approximately 50% when tested at 0.1 and 1 μM. These results were translated *in vivo* whereby administration of GW9508 (10 mg/kg, i.p.) during *E. coli* infections resulted in elevated peritoneal leukocyte infiltration with a higher phagocytic capacity. Importantly, GW9508 administration also modulated the lipid mediator profile, with increased levels of the pro-resolving mediators resolvin D3 and lipoxins. In conclusion, GPR40 is expressed by activated neutrophils and plays an important host protective role to aid clearance of bacterial infections.

## Introduction

G-protein-coupled receptor 40 (GPR40; also known as free fatty acid receptor 1, FFAR1), is a member of the G-protein coupled receptor (GPCR) family. Many agonists have been discovered to bind and activate GPR40 such as medium- and long-chain fatty acids, including omega-3 docosahexaenoic acid (DHA) (1) as well as 17,18-epoxyeicosatetraenoic acid (17,18-EpETE), a bioactive lipid mediator (LM) derived from eicosapentaenoic acid (EPA) (2). G-protein-coupled receptor 40 is highly expressed in pancreatic beta cells, where it is involved in the regulation of insulin secretion (3). Indeed, due to its pivotal role in insulin regulation a number of synthetic agonists have been developed such as Fasiglifam (TAK-875) and GW9508, which exert beneficial effects in diabetes (4, 5). Importantly, there is evidence that GPR40 also plays a role in regulating the inflammatory response, for example by counteracting inflammasome activation and limiting contact hypersensitivity (6, 2). However, the functional role of GPR40 in the context of the innate immune response to infection and whether it plays a role in the resolution of inflammation remains to be fully elucidated.

In the present study, we demonstrate that GPR40 expression is up-regulated on human neutrophils under inflammatory settings. Using *in vitro* assays, we demonstrated that GW9508-stimulation induces calcium mobilization, increases neutrophil chemotaxis toward IL-8 and enhances bacterial phagocytosis. In an acute model of *Escherichia coli* infection, GW9508 improved clearance of *E. coli* by peritoneal leukocytes and reprogrammed the LM expression toward a more pro-resolution profile. Our work identified a previously unknown role of GPR40 in enhancing neutrophil responses, which is important for maintaining host defense against pathogens.

## Materials and Methods

### Blood Collection

Volunteers gave written consent in accordance with a Queen Mary Research Ethics Committee (QMREC 2014:61). Venous peripheral blood was collected from healthy volunteers into sodium citrate (3.2%), and neutrophils were isolated using dextran sedimentation followed by gradient centrifugation.

### Collection of Exudated Human Neutrophils

A model of acute neurogenic inflammation was performed to collect activated human neutrophils from the oral cavity according to a protocol approved by the Queen Mary Research Ethics Committee (QMREC2010/17). Volunteers were asked to rinse the buccal cavity three times with 20 ml of 0.9% saline for 30 s, followed by a 10% Tabasco^®^ solution (20 ml for 30 s). The volunteers were *nil by mouth* for the following 2 h, prior to rinsing the buccal cavity again three times with 20 ml of 0.9% saline (7). Mouthwash samples were collected, passed through a 70 μm strainer and centrifuged at 300 *g* for 10 min at room temperature. Cells were washed with 50 ml of DPBS^–/–^, passed through a 40 μm strainer to remove epithelial cells and centrifuged at 300 *g* for 10 min at room temperature. The supernatant was discarded and the cells were gently re-suspended for further analysis.

### Flow Cytometry

Neutrophils were stimulated with vehicle (0.1% ethanol), TNF-α (10 ng/ml), IL-8 (10ng/ml), platelet-activating factor (PAF; 10 nM), or leukotriene B_4_ (LTB_4_; 10 nM) for 10 min at 37°C prior to analysis of GPR40 expression. Cells were fixed and permeabilized according to manufacturer’s instructions (eBioscience), then incubated with anti-GPR40 (0.181 μg/ml, clone EP4632; Abcam) for 30 min on ice, washed three times and a goat anti-rabbit secondary antibody (AlexaFluor 488, Life Technologies) was added for 45 min on ice. G-protein-coupled receptor 40 expression was recorded as MFI units in the FL1 channel of a BD FACSCalibur or in the B530/30 channel of a BD LSR Fortessa.

### ImageStream Analysis

Cells were incubated with APC-anti-CD11b (clone ICRF44; eBioscience) and PE-Cy5-anti-CD62L (clone DREG56; eBioscience) for 45 min at 4°C in DPBS containing 0.02% BSA. After staining, red blood cells were lysed using Whole Blood Lysing Reagent Kit, according to the manufacturer’s instructions. Staining was then assessed using ImageStream X MK2 and analysis was performed using IDEAS^®^ (Image Data Exploration and Analysis Software, Version 6.0).

### Intracellular Calcium Mobilization

Human neutrophils were incubated with 2 μM Fura 2-AM (Molecular Probes, Paisley, United Kingdom) in HBSS without Ca^2+^ (Sigma-Aldrich) at 37°C for 45 min in the dark then washed three times with HBSS. HBSS containing 0.185 g/l CaCl_2_ was then added before stimulation with GW9508 (0–10 μM) or Ionomycin (1 μM). Mobilization of intracellular calcium was measured for 70 s after treatment by recording the ratio of fluorescence emission at 510 nm after sequential excitation at 340 and 380 nm using the NOVOstar microplate reader (BMG LABTECH, Aylesbury, United Kingdom). The results are expressed as percentage of the positive control (ionomycin) or as delta of time zero.

### Chemotaxis Assay

Human neutrophils were stimulated with GW9508 (0.1–10 μM) or vehicle for 10 min at 37°C. Chemotaxis was performed using 3-μm pore size ChemoTx^TM^ 96 well plates (Neuro Probe Inc, Gaithersburg, United States) (8) for 90 min. Briefly, migrated cells were collected from the bottom chamber and incubated with PrestoBlue^®^ (Invitrogen Ltd., Paisley, United Kingdom) and compared with a standard curve constructed with known cell numbers. Plates were read after 4 h in a fluorescence spectrophotometer at EX560-EM590 nm.

### Phagocytosis Assay

Human neutrophils were stimulated with GW9508 (0.1–10 μM) for 10 min at 37°C in RPMI containing 0.1% FBS. After treatment, BODIPY (576/589)-labeled *E. coli* (1 mg/ml) was added for 30 min at 37°C, 5% CO_2_ and then neutrophils were washed three times with cold DPBS to remove bacteria that had not been phagocytosed. Phagocytosis levels were determined using a fluorescence plate reader and are expressed as fluorescence intensity or as the percentage of the positive control.

### Apoptosis

Human neutrophils were stimulated with GW9508 (10 μM) or vehicle and incubated at 37°C in a 5% CO_2_ incubator. After 2, 8, 18 and 24 h of incubation, neutrophils were loaded in cytospin chambers, fixed in methanol and stained with H&E. About 200 cells per slide were counted with ×100 objective. In another set of experiments, apoptosis was assessed by flow cytometry with the Dead Cell Apoptosis Kit according to manufacturer’s instructions. Briefly, after 18 h incubation, neutrophils (1 × 10^5^) were washed twice and resuspended in 1× binding buffer, followed by the addition of Annexin V FITC and PI for 15 min at room temperature in the dark. Samples were analyzed within 1 h and AnxV binding and PI staining was recorded as MFI units in the B530/30 and YG610/20 channels respectively, using a BD LSR Fortessa.

### Animals

Male C57BL/6 mice (8 weeks old) were procured from Charles River (Kent, United Kingdom). Experiments strictly adhered to UK Home Office regulations (Scientific Procedures Act, 1986) and Laboratory Animal Science Association (LASA) Guidelines. All animals were provided with standard laboratory diet and water *ad libitum* and kept on a 12 h light/dark cycle.

### Peritonitis

*Escherichia coli* (serotype O6:K2:H1) were cultured in LB broth and harvested at mid-log phase (OD600 ∼0.5, 5 × 10^8^ CFU/ml) and washed in sterile saline before inoculation into the mouse peritoneum. Mice were given live *E. coli* (1 × 10^5^) i.p. and treated with GW9508 (10 mg/kg 100 μl, i.p.) or vehicle 1 h later. After 12 h, mice were euthanized and peritoneal exudates and blood were collected. Leukocyte infiltration to the peritoneum was assessed using Ly6G PE (clone 1A8, eBioscience) for neutrophils, Ly6C eFluor450 (clone HK1.4, eBioscience) for monocytes and F4/80 BV650 (clone: BM8, eBioscience) for macrophages. Phagocytosis of *E. coli* was determined following cell permeabilization and staining with FITC-conjugated *E. coli* antibody (GeneTex).

### Targeted Lipid Mediator Profiling

All samples for LC-MS-MS-based profiling were extracted using solid-phase extraction columns (9). Three microliter of peritoneal exudate were placed in ice-cold methanol containing deuterated internal standards, representing each region in the chromatographic analysis (500 pg each). Samples were kept at -20°C for 45 min to allow protein precipitation. Supernatants were subjected to solid phase extraction, methyl formate fraction collected, brought to dryness and suspended in phase (methanol/water, 1:1, vol/vol) for injection on a Shimadzu LC-20AD HPLC and a Shimadzu SIL-20AC autoinjector, paired with a QTrap 5500 (Sciex). An Agilent Poroshell 120 EC-C18 column (100 mm × 4.6 mm × 2.7 μm) was kept at 50°C and mediators eluted using a mobile phase consisting of methanolwater-acetic acid of 20:80:0.01 (vol/vol/vol) that was ramped to 50:50:0.01 (vol/vol/vol) over 0.5 min and then to 80:20:0.01 (vol/vol/vol) from 2 min to 11 min, maintained till 14.5 min and then rapidly ramped to 98:2:0.01 (vol/vol/vol) for the next 0.1 min. This was subsequently maintained at 98:2:0.01 (vol/vol/vol) for 5.4 min, and the flow rate was maintained at 0.5 ml/min. The QTrap 5500 was operated using a multiple reaction monitoring method. Each LM was identified using established criteria including matching retention time to synthetic and authentic materials and at least six diagnostic ions (9).

### Statistical Analysis

Results are presented as mean ± SEM. Differences between groups were assessed using GraphPad Prism 7 (GraphPad Software, La Jolla, United States) and 1-way ANOVA with *post hoc* Dunnett’s or Student’s *t*-test. The criterion for statistical significance was *p* < 0.05. Partial least squares-discrimination analysis (PLS-DA) and principal component analysis (PCA) (10) were performed using SIMCA 14.1 software 6 (Umetrics, Umea, Sweden) following mean centering and unit variance scaling of LM levels. Partial least squares-discrimination analysis is based on a linear multivariate model that identifies variables that contribute to class separation of observations (peritoneal exudates) on the basis of their variables (LM levels). During classification, observations were projected onto their respective class model. The score plot illustrates the systematic clusters among the observations (closer plots presenting higher similarity in the data matrix). Loading plot interpretation identified the variables with the best discriminatory power (Variable Importance in Projection greater than 1) that were associated with the distinct intervals and contributed to the tight clusters observed in the Score plot.

## Results

### GPR40 Expression Is Upregulated on Activated Human Neutrophils

**FIGURE 1:**
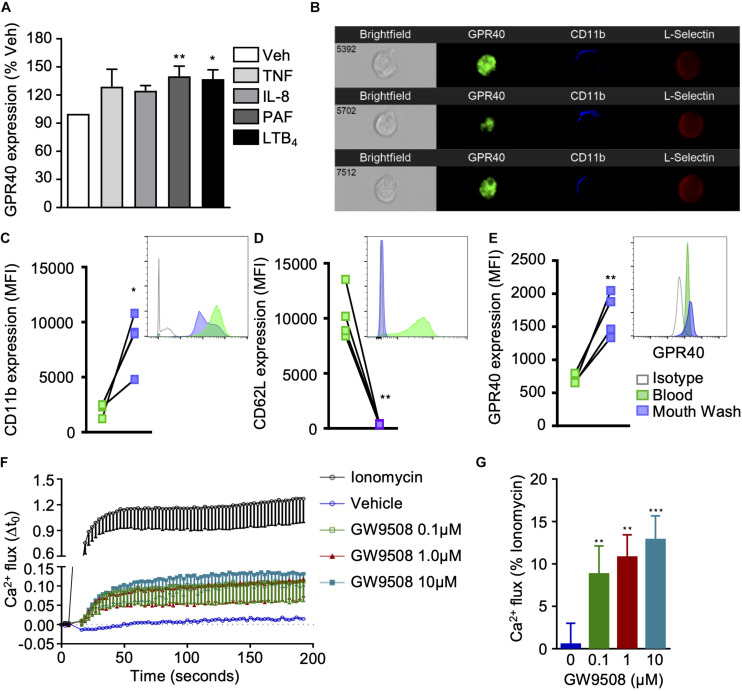
GPR40 expression and agonist activation in human neutrophils. **(A)** Neutrophils isolated from healthy volunteers were stimulated with vehicle (0.1% ethanol), TNF-α (10 ng/ml), IL-8 (10 ng/ml), PAF (10 nM) or LTB_4_ (10 nM) for 10 min at 37°C, and GPR40 expression monitored by flow cytometry. Results are mean ± SEM, *n* = 4 *p < 0.05 and **p < 0.01 vs. vehicle control using one-way ANOVA, followed by Dunnett’s post-test. **(B)** Representative images of GPR40 expression in unstimulated neutrophils (CD11b^low^CD62L^hi^) by Imagestream^TX^ (60x). **(C–E)** Neutrophils were isolated from the peripheral blood and from the buccal cavity after mouth wash with Tabasco^TM^ from healthy individuals and expression of **(C)** CD11b, **(D)** L-selectin, and **(E)** GPR40 was monitored by flow cytometry, representative histograms are shown inset. Results are mean ± SEM, *n* = 4. *p < 0.05, **p < 0.01 compared to peripheral blood using a paired *T*-test. **(F,G)** Isolated neutrophils were incubated with Fura 2-AM and treated with vehicle control, ionomycin (positive control) or GW9508 (0.1, 1, and 10 μM), and calcium flux was monitored over time. **(G)** Intracellular calcium flux expressed as a percentage of the maximal response induced by ionomycin. Results are expressed as mean ± SEM from four independent experiments. **p < 0.01 and ***p < 0.001 compared to vehicle (0.1% ethanol); 1-way ANOVA, followed by Bonferroni post-test.

We first assessed whether GPR40 was expressed by human neutrophils and whether it could be differentially modulated following cell activation. To mimic inflammatory settings, neutrophils were stimulated with TNF-α, IL-8, PAF, or LTB_4_ for 10 min and GPR40 levels were analyzed by flow cytometry. G-protein-coupled receptor 40 was moderately increased by TNF-α (10 ng/ml) and IL-8 (10 ng/ml) when compared to vehicle (0.1% ethanol) treated cells (Figure 1A). Whereas PAF (10 nM) and LTB_4_ (10 nM) stimulation significantly increased GPR40 levels (Figure 1A). G-protein-coupled receptor 40 expression was also visualized by imaging flow cytometry, which demonstrated localization throughout the cell in resting neutrophils characterized by low CD11b and high L-selectin surface levels (Figure 1B).

It is well known that neutrophil recruitment to the site of inflammation results in the activation of these adhesion molecules. The sensing of chemokines, and the physical contact with endothelial cells promotes a change in neutrophil phenotype, with substantial alterations in cellular composition, due to release of secretory vesicles and granules (11). Thus, a model of acute neurogenic inflammation was performed to collect activated human neutrophils from the oral cavity. As expected, neutrophils freshly isolated from whole blood exhibited basal expression levels of CD11b, whereas significantly higher levels were detected on extravasated neutrophils, promoted by tabasco mouth wash (Figure 1C). Conversely, blood neutrophils expressed high levels of L-selectin that was shed during recruitment (Figure 1D). Interestingly, GPR40 expression was significantly higher on exudate neutrophils when compared to neutrophils isolated from peripheral blood of the same individual, further confirming that activated neutrophils express higher GPR40 levels (Figure 1E).

Next, it was essential to demonstrate that GPR40 was functional on human neutrophils, thus GW9508, a selective GPR40 agonist, was tested. Previous studies investigating GPR40 signaling in pancreatic islet cells have eluded that this GPCR is coupled to the α subunit of the Gq family of G proteins, leading to PLC activation, hydrolysis of inositol lipids and increased intracellular calcium levels (12). Therefore, we measured the intracellular calcium flux in human neutrophils treated with a concentration range (0.1–10 μM) of GW9508, or ionomycin as a positive control. At all concentrations tested, GW9508 promoted an intracellular calcium flux that was significantly greater than the vehicle control (Figures 1F,G).

### GW9508 Enhances Neutrophil Functionality

**FIGURE 2:**
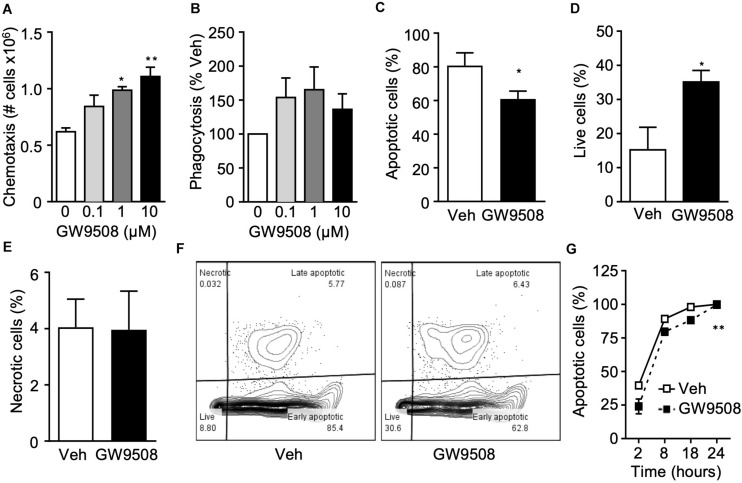
GW9508 enhances human neutrophil survival and function. **(A)** Human neutrophils were isolated from healthy volunteers and treated with GW9508 (0.1–10 μM) or vehicle (0.1% ethanol) for 10min at 37°C and chemotaxis to IL-8 (30 ng/ml, 1 h) was assessed. Results are expressed as mean ± SEM from four independent experiments. **(B)** GW9508 treated neutrophils were incubated with BODIPY-labeled *E. coli* (30 min, 37°C) and phagocytosis was assessed by fluorescence. Results are expressed as percent increase above vehicle, mean ± SEM from five independent experiments. **(C)** Human neutrophils were treated with GW9508 (10μM), and were cultured overnight to allow spontaneous apoptosis. After 18 h, annexin V binding and PI staining was assessed by flow cytometry for quantification of **(C)** apoptotic (AnxV^+^PI^–^ ), **(D)** live (AnxV^–^ PI^–^ ), and **(E)** necrotic (AnxV^–^ PI^+^) cells. Results are expressed as mean ± SEM from three independent experiments. *p < 0.05 compared to Veh; Unpaired T-test. **(H)** A time-course of neutrophil apoptosis was performed by assessing nuclear condensation by light microscopy following H&E staining. Results are expressed as mean ± SEM from three independent experiments. **p < 0.01 compared to Veh; 2-way ANOVA, followed by Bonferroni post-test.

Since GPR40 was upregulated on activated neutrophils and GW9508 treatment induced intracellular signaling, we next investigated the functional significance of the GPR40-GW9508 axis on neutrophil reactivity. Thus, the effects of GW9508 on neutrophil chemotaxis in response to the chemokine IL-8 were assessed. Isolated human neutrophils were incubated with vehicle (0.1% ethanol) or pre-incubated with GW9508 (0.1, 1, and 10 μM) for 10 min, and the migratory response to IL-8 tested. Incubation of neutrophils with GW9508 enhanced the chemotactic response compared with vehicle alone. This effect was concentration-dependent, with the highest concentration of 10 μM evoking an 80% increase in cell migration compared with IL-8 alone (Figure 2A).

One of the major functions of neutrophils is to safely and efficiently clear bacteria and cellular debris, to help bring the tissue back to homeostasis, a key step in the resolution of inflammation. Thus, we next investigated whether GW9508 could alter the phagocytic ability of neutrophils. Phagocytosis was determined after incubation for 90 min with fluorescently labeled *E. coli*. Neutrophils treated with GW9508 had an increased phagocytic capacity, as determined by the amount of intracellular *E. coli*. Indeed, 0.1 μM GW9508 increased neutrophil phagocytosis by approximately 50% when compared to vehicle. The optimal concentration of GW9508 was 1 μM, leading to enhanced phagocytosis of 60% over vehicle treatment (Figure 2B).

After neutrophils have killed pathogens and cleared debris it is essential that they undergo apoptosis, a process of controlled cell death necessary for their safe removal from an inflammatory site. Cell death by necrosis, on the other hand can cause tissue damage by release of harmful substances such as reactive oxygen species (ROS) and proteases following rupture of the cell membrane. Therefore, the effects of GW9508 on neutrophil cell death were determined by measuring annexin V binding and propidium iodide (PI) staining after culturing overnight in RPMI containing 0.1% FBS (18 h). Surprisingly, GW9508 decreased the number of apoptotic cells (Figure 2C) and enhanced neutrophil survival (Figure 2D). Importantly, GW9508 did not induce cellular necrosis as determined by the percentage of AnxV^–^PI^+^ cells (Figure 2E), with representative flow cytometry plots shown in Figure 2F. We therefore performed a full time-course of neutrophil apoptosis to determine whether GW9508 would prolong the lifespan of neutrophils (Figure 2G). GW9508 prevented neutrophil apoptosis as early as 2 h after stimulation, an effect that was observed up to 18 h after treatment (Figure 2G). Yet almost 100% of neutrophils were apoptotic by 24 h, with or without treatment, suggesting that the effects of GW9508 are temporal. GW9508 treatment had no significant impact on the clearance of apoptotic PMN via the process of efferocytosis (data not shown).

### GW9508 Enhances Leukocyte Recruitment and Bacterial Clearance *in vivo*

**FIGURE 3:**
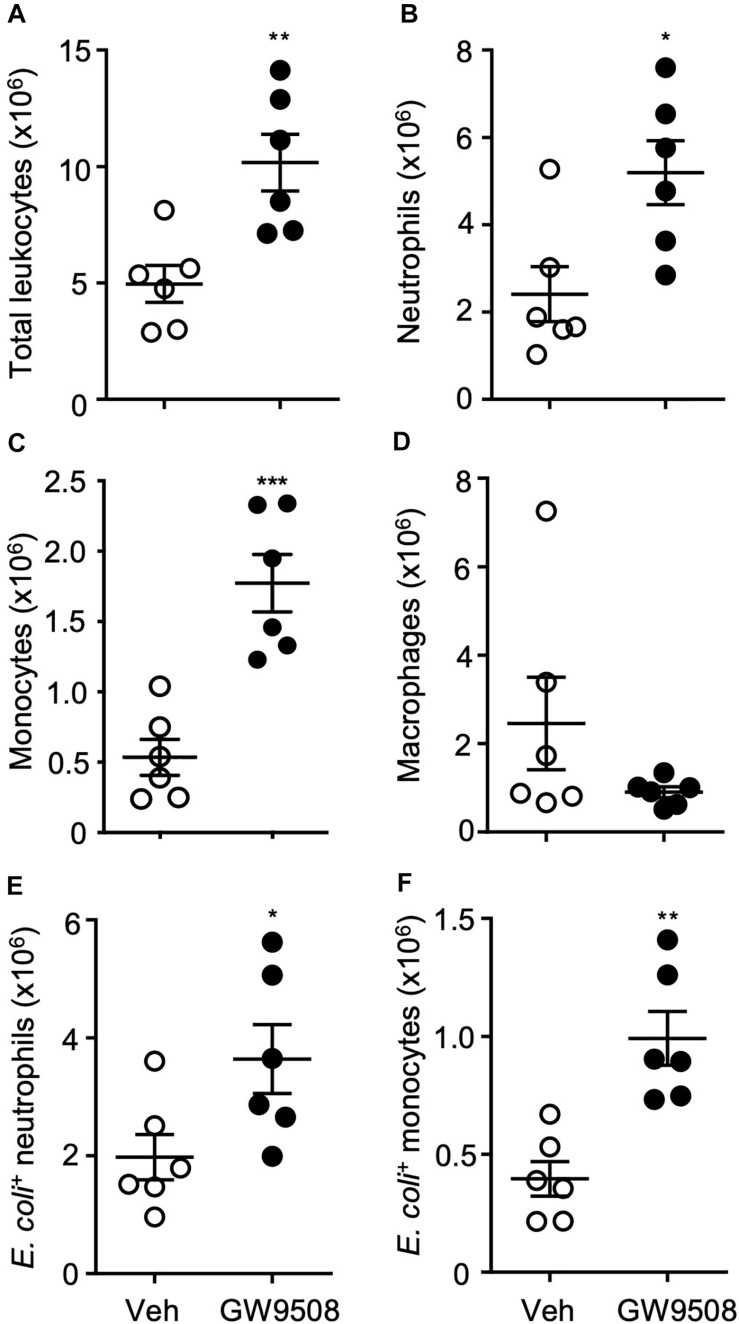
GW9508 increases leukocyte recruitment and bacterial clearance. Mice were administered GW9508 (10 mg/kg/mouse) or vehicle (PBS) and 1h later inoculated with *E. coli*. (10^5^ i.p.). Peritoneal exudates were collected after 12 h and **(A)** total leukocyte recruitment, **(B)** neutrophils, **(C)** monocytes, and **(D)** macrophages were enumerated. Phagocytic clearance of *E.coli* was quantified by flow cytometry by assessing the number of positive **(E)** neutrophils and **(F)** monocytes. Results are expressed as mean ± SEM, *n* = 6 mice per group. *p < 0.05, **p < 0.01 compared to vehicle using an unpaired *t*-test.

Next, we questioned whether the chemotactic and phagocytic properties of GW9508 visualized *in vitro* would remain *in vivo*. Mice were inoculated with live *E.coli* (10^5^) i.p. to induce peritonitis, followed by GW9508 (10 mg/kg/mouse) or vehicle control (0.1%PBS) 1h later, and mice were sacrificed after 12 h at peak neutrophil infiltration (13). Peritoneal exudates of GW9508 treated mice contained an increased number of total leukocytes (Figure 3A), more specifically neutrophils and monocytes, compared to vehicle-treated mice (Figures 3B,C). Macrophage numbers were not significantly altered at this time point (Figure 3D). Importantly, GW9508-treatment led to increased numbers of *E.coli* positive neutrophils and monocytes (Figures 3E,F) compared to vehicle-treated mice, indicating enhanced containment and clearance of bacteria.

### GW9508 Stimulates Pro-resolving Lipid Mediators During Peritonitis

**FIGURE 4:**
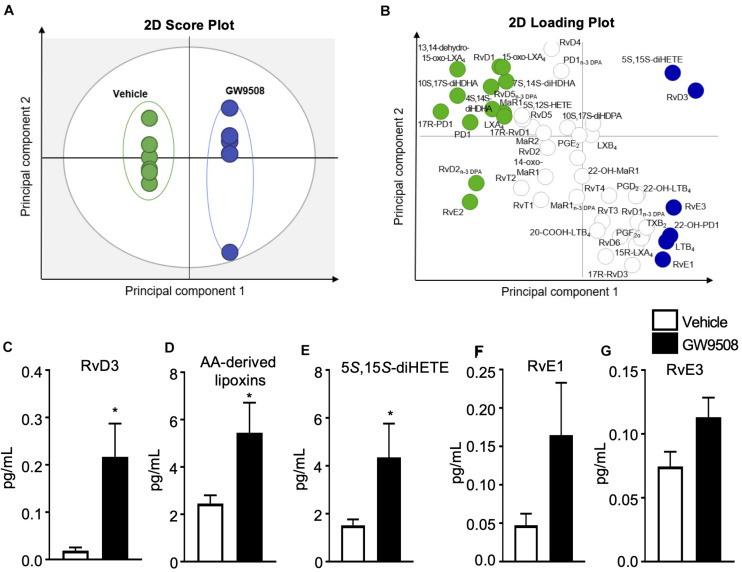
GW9508 induces SPM production in *E. coli* infected mice. Mice were administered GW9508 (10 mg/kg/mouse) or vehicle (PBS) and 1h later inoculated with *E. coli*. (10^5^ i.p.). Peritoneal exudates were collected after 12 h and lipid mediators were identified and quantified by using liquid chromatography tandem mass spectrometry (LC-MS/MS) lipid mediator profiling and exudate lipid mediator concentrations were assessed using panels **(A,B)**. Partial least squares discriminant analysis **(A)** 2-dimensional (2D) score plot of the distinct LM-SPM profiles identified in the different treatment groups, **(B)** corresponding 2D loading plot. Gray ellipse in the score plots denotes 95% confidence regions. Green and blue ellipses represent LM with a variable in importance score ≥ 1; *n* = 5–6 mice per group. **(C)** RvD3, **(D)** AA-derived lipoxins, **(E)** 5S,15S-diHETE, **(F)** RvE1, and **(G)** RvE3 concentrations identified and quantified in vehicle and GW9508 treated mice. Results are expressed as pg/mL and indicate the mean ± SEM, *n* = 5–6 mice per group, *p ≤ 0.05 vs. vehicle using an unpaired *t*-test.

Given the host protective actions of GW9508 during *E.coli* infection, we next determined whether this response was associated with a pro-resolving signature by assessing the LM profile of the peritoneal exudates. Lipid mediators were identified and quantified by using liquid chromatography-tandem mass spectrometry-based LM profiling. The identity of lipid mediators was ascertained in accordance with published criteria, that included matching retention times to authentic or synthetic standards and identification of at least 6 diagnostic ions in the tandem mass spectrometry (MS-MS) fragmentation spectrum (9). In these inflammatory exudates we identified mediators from all four essential fatty acid metabolomes, including D-series resolvins and lipoxins (Supplementary Table 1). Of note, the concentrations of these mediators were within their described bioactive ranges (14). Multivariate analysis of peritoneal exudate LM profiles, demonstrated two distinct clusters representing LM profiles obtained from vehicle- and GW9508-treated mice (Figures 4A,B). GW9508 treatment was associated with significantly increased levels of RvD3 and AA-derived lipoxins (Figures 4C,D). In addition, there was a 2-fold increase in the lipoxin pathway marker 5S,15S-diHETE (Figure 4E), a 3-fold increase in the levels of RvE1 and increased levels of RvE3 (Figures 4F,G) in peritoneal exudates from GW9508-treated mice compared to vehicle control.

## Discussion

Our data herein substantiates an important role for GPR40 in the host inflammatory response to curtail and contain bacterial infection. We made the novel observation that GPR40 is upregulated on human neutrophils upon activation with a variety of pro-inflammatory substances and following extravasation into the oral cavity in response to an inflammatory challenge. We utilized the well characterized GPR40 synthetic agonist GW9508 to elucidate the downstream actions of GPR40 stimulation in human neutrophils. We found that GW9508 could enhance chemotaxis and temporarily prolong neutrophil lifespan, which we believe may be a mechanism to aid the timely clearance of bacteria. When tested in a self-limited infection model, GW9508 accelerated the resolution of *E. coli* infection by increasing leukocyte recruitment, phagocytic clearance of bacteria and stimulating certain pro-resolving lipid mediators.

Neutrophils and their armamentarium contribute to initiation, development and resolution of the inflammatory response (15). Thus, control of when, where and how neutrophils act must be tightly regulated to maintain a healthy immune system. Neutrophil effector functions are regulated via a vast variety of receptors, some of which are contained in intracellular granules that can be rapidly mobilized to the cell surface upon neutrophil activation (11). Indeed, the phenotype of extravasated neutrophils is known to be significantly modulated compared with those circulating within the vasculature (16). In the oral cavity, it has been reported that neutrophils elicited following capsaicin challenge are primed to produce significantly more ROS than resident neutrophils prior to challenge or peripheral blood neutrophils from the same donor (7). We report here that neutrophils migrating into the oral cavity in response to capsaicin have an activated phenotype with high levels of CD11b, minimal L-selectin levels and significantly higher levels of GPR40. This finding corroborates our *in vitro* experiments whereby the pro-inflammatory lipid mediators PAF and LTB_4_ upregulated GPR40 expression on isolated peripheral blood neutrophils. It is worth noting that elevated levels of GPR40 are also detected on renal epithelial cells in models of kidney fibrosis including unilateral ureteral obstruction, ischemic injury, and adenine-induced nephropathy, where it is deemed protective (17), thus suggesting that this receptor may be upregulated within inflammatory settings to assist, we propose, in resolution and tissue-reparative mechanisms. Notably, other pro-resolving receptors such as ChemR23 and FPR2/ALX are elevated on the cell surface of PMN following activation with inflammogens such as TNF-α and IL-8 (18, 19) as well as recruitment to human blisters (20), further supporting the concept that pro-resolving receptors can be rapidly mobilized to the cell membrane to counter regulate inflammation.

G-protein coupled receptors are promiscuous both in terms of agonist activation as well as interaction with binding partners (21). It is well known that GPR40 can be activated by various medium and long chain free fatty acids, often producing opposing actions (1). In this work, we focused on the use of GW9508, a synthetic agonist proven to be beneficial in diabetes, to elucidate whether the GPR40 pathway would be protective in the context of bacterial infection. Rapid recruitment of neutrophils and efficient chemotaxis to sites of infection are essential preludes to neutrophil function and clearance of bacteria. Intriguingly, GW9508 treatment can induce IL-8 release from bovine neutrophils (22). This chemokine is a powerful attractant for both neutrophils and monocytes and may explain why higher numbers of these leukocytes are recruited to the peritoneal cavity following GW9508 administration (Figure 3) in a feed-forward mechanism.

Depending on the agonist and environmental conditions, GPR40 signaling can either induce or protect from cellular apoptosis. Similarly to our findings with human neutrophils (Figure 2), GW9508 attenuated apoptosis of human renal epithelial cells in an injury model. The mechanism behind these protective actions included inhibition of reactive oxygen species (ROS) generation, pro-apoptotic proteins and nuclear factor-κB (NF-κB) activation (23). Further studies are required to elucidate the mechanism by which GW9508 delays the spontaneous apoptosis of human neutrophils.

Bacterial peritonitis caused by *E. coli* infection is a clinically important problem with a high mortality rate (24). If infection is not contained and eliminated by phagocytes it can rapidly progress leading to excessive inflammation, epithelial and endothelial barrier dysfunction, immune suppression and multiple-organ failure that can be deadly. Thus, timely clearance of bacteria is essential. Importantly, we found that administration of GW9508 could enhance phagocytic clearance of *E.coli* from the peritoneum. Interestingly, another GPR40 agonist has been documented to prevent bacterial dissemination by inhibiting epithelial barrier impairment induced by the periodontopathic bacterium *Porphyromonas gingivalis*. This endogenous agonist is a bioactive metabolite generated by probiotic microorganisms during the process of fatty acid metabolism known as 10-hydroxy-cis-12-octadecenoic acid (HYA), which signals via GPR40 on gingival epithelial cells to exert its beneficial actions (25). We have previously reported that alpha-2-macroglobulin loaded microparticles enhance host responses to infection by promoting neutrophil recruitment and clearance of bacteria whilst stimulating pro-resolving pathways (26), thus promoting a swift resolution of bacterial sepsis. Using a human blister model to investigate inflammation-resolution, Morris et al., reported that two types of responders exist, those with immediate leukocyte accumulation followed by early resolution and those with delayed resolution. Timely resolution of cantharidin-induced skin blisters was due at least in part to endogenous levels of 15epi-LXA_4_ and its receptor ALX/FPR2 expression (20). We therefore deem the enhanced leukocyte recruitment observed with GW9508 treatment in *E. coli* peritonitis to be a protective response to prevent the unwanted spread of bacteria.

One of the mechanisms by which the GPR40 agonist GW9508 aided bacterial clearance was *via* regulation of specific specialized pro-resolving lipid mediators. These mediators derived from omega-3 and omega-6 fatty acids are known to stimulate phagocyte functions to control bacterial infections and accelerate the host immune response to infection (13, 27). Whilst we found that specific lipid mediators were elevated in response to the GPR40 agonist: these were RvD3, lipoxins, RvE1 and RvE3 (Figure 4). Notably, RvD3, lipoxins and RvE1 are effective in enhancing the clearance of *E. coli* infection (28), bacterial pneumonia (29), and resolution of UV-killed *E. coli* in human blisters (30). Systematic analysis of pro-resolving lipid mediator profiles in septic patients with acute respiratory distress syndrome (ARDS) indicated that the amount of circulating 10*S*,17*S*-diHDHA (PDX) at day 3 was a better predictor of ARDS development than the APACHE II score (31), further supporting the role of SPM in regulating host responses during infections.

Together, our study indicates receptor-mediated actions of GW9508, with direct regulation of neutrophil function to enhance clearance of *E. coli*. We propose that GPR40 activation could be beneficial in infection not only through regulation of neutrophil responses but also through exquisite regulation of lipid mediators. Whether these effects are restricted to GW9508 and agonists which may behave in a similar fashion remains to be elucidated. In any case, uncovering new therapeutics that aid in the timely resolution of infection is imperative to prevent bacterial dissemination that could lead to unwanted organ damage and life-threatening conditions, and GPR40 could be explored to enable this long-term objective.

## Data Availability Statement

The raw data supporting the conclusions of this article will be made available by the authors, without undue reservation.

## Ethics Statement

The studies involving human participants were reviewed and approved by Queen Mary Research Ethics Committee. The patients/participants provided their written informed consent to participate in this study. The animal study was reviewed and approved by UK Home Office.

## Author Contributions

PS designed and performed *in vitro* and *in vivo* experiments and wrote the manuscript. MW performed lipid mediator profiling analysis and contributed to the manuscript. NG designed human mouthwash model. JD designed *in vivo* experiments and contributed to the manuscript. MP coordinated the project and wrote the manuscript. LN coordinated the project, performed *in vitro* experiments and wrote the manuscript. All authors contributed to the article and approved the submitted version.

## Conflict of Interest

The authors declare that the research was conducted in the absence of any commercial or financial relationships that could be construed as a potential conflict of interest.
