# Supplementary material for: The GPR40 Agonist GW9508 Enhances Neutrophil Function to Aid Bacterial Clearance During E. coli Infections
Source: Front Immunol. 2020 Sep 29;11:573019. doi: 10.3389/fimmu.2020.573019 (PMC7550532; doi:10.3389/fimmu.2020.573019)
Supplement: Supplementary file 1 [file Table_1.DOCX]

**Supplementary Table 1: Peritoneal lipid mediator concentrations during self-limited *E.coli* peritonitis.** Results are mean ± SEM, n=5 per group and expressed as pg/mL. *p≤0.05, **p≤0.01 and ***p≤0.001 vs vehicle using unpaired t-test.

| pg/mL lavage | Q1 | Q3 | Vehicle | | | Agonist | | |
| --- | --- | --- | --- | --- | --- | --- | --- | --- |
|  |  |  | Mean | ± | SEM | Mean | ± | SEM |
| **DHA-derived metabolome** |  |  |  |  |  |  |  |  |
| RvD1 | 375 | 233 | 0.22 | ± | 0.02 | 0.17 | ± | 0.03 |
| RvD2 | 375 | 215 | 0.21 | ± | 0.04 | 0.18 | ± | 0.02 |
| RvD3 | 375 | 147 | 0.02 | ± | 0.01 | 0.22 | ± | 0.07 ** |
| RvD4 | 375 | 225 | 0.03 | ± | 0.01 | 0.02 | ± | 0.01 |
| RvD5 | 359 | 199 | 0.32 | ± | 0.18 | 0.11 | ± | 0.04 |
| RvD6 | 359 | 159 | 0.05 | ± | 0.01 | 0.06 | ± | 0.02 |
| 17*R*-RvD1 | 375 | 215 | 0.04 | ± | 0.01 | 0.03 | ± | 0.00 |
| 17*R*-RvD3 | 375 | 147 | 0.01 | ± | 0.00 | 0.10 | ± | 0.10 |
| PD1 | 359 | 153 | 0.37 | ± | 0.09 | 0.12 | ± | 0.02 ** |
| 17*R*-PD1 | 359 | 153 | 0.48 | ± | 0.10 | 0.02 | ± | 0.01 *** |
| 10*S*,17*S*-diHDHA | 359 | 153 | 5.40 | ± | 1.50 | 0.32 | ± | 0.03 *** |
| 22-OH-PD1 | 375 | 153 | 0.03 | ± | 0.00 | 0.05 | ± | 0.01 * |
| MaR1 | 359 | 177 | 0.38 | ± | 0.11 | 0.19 | ± | 0.04 |
| 7*S*,14*S*-diHDHA | 359 | 177 | 0.29 | ± | 0.10 | 0.12 | ± | 0.02 |
| MaR2 | 359 | 191 | 0.23 | ± | 0.10 | 0.15 | ± | 0.04 |
| 4,14-diHDHA | 359 | 159 | 0.63 | ± | 0.22 | 0.18 | ± | 0.04 * |
| 22-OH-MaR1 | 375 | 221 | 0.23 | ± | 0.03 | 0.23 | ± | 0.06 |
| 14-oxo-MaR1 | 375 | 248 | 0.13 | ± | 0.04 | 0.10 | ± | 0.02 |
| **DPA-derived metabolome** |  |  |  |  |  |  |  |  |
| RvT1 | 377 | 211 | 1.32 | ± | 0.18 | 0.52 | ± | 0.15 *** |
| RvT2 | 377 | 197 | 0.08 | ± | 0.02 | 0.05 | ± | 0.01 |
| RvT3 | 377 | 173 | 0.04 | ± | 0.00 | 0.04 | ± | 0.01 |
| RvT4 | 361 | 211 | 0.06 | ± | 0.02 | 0.07 | ± | 0.03 |
| RvD1_n-3 DPA_ | 377 | 215 | 0.04 | ± | 0.01 | 0.06 | ± | 0.02 |
| RvD2_n-3 DPA_ | 377 | 261 | 0.09 | ± | 0.01 | 0.05 | ± | 0.01 * |
| RvD5_n-3DPA_ | 377 | 199 | 0.10 | ± | 0.02 | 0.06 | ± | 0.01 * |
| PD1_n-3 DPA_ | 361 | 183 | 0.15 | ± | 0.02 | 0.13 | ± | 0.04 |
| 10*S*,17*S*-diHDPA | 361 | 155 | 0.12 | ± | 0.04 | 0.10 | ± | 0.03 |
| MaR1_n-3 DPA_ | 361 | 223 | 0.05 | ± | 0.01 | 0.05 | ± | 0.01 |
| 7*S*,14*S*-diHDPA | 361 | 223 | 0.07 | ± | 0.01 | 0.09 | ± | 0.04 |
| **EPA-derived metabolome** |  |  |  |  |  |  |  |  |
| RvE1 | 349 | 195 | 0.05 | ± | 0.02 | 0.16 | ± | 0.07 |
| RvE2 | 333 | 159 | 0.14 | ± | 0.02 | 0.06 | ± | 0.02 ** |
| RvE3 | 333 | 201 | 0.07 | ± | 0.01 | 0.11 | ± | 0.02 * |
| **AA-derived metabolome** |  |  |  |  |  |  |  |  |
| LXA_4_ | 351 | 115 | 0.04 | ± | 0.01 | 0.03 | ± | 0.00 |
| LXB_4_ | 351 | 221 | 0.18 | ± | 0.08 | 0.19 | ± | 0.04 |
| 5*S*,15*S*-diHETE | 335 | 235 | 1.51 | ± | 0.25 | 4.35 | ± | 1.42 * |
| 15-epi-LXA_4_ | 351 | 115 | 0.13 | ± | 0.03 | 0.31 | ± | 0.16 |
| 15-epi-LXB_4_ | 351 | 115 | 0.16 | ± | 0.05 | 0.28 | ± | 0.09 |
| 13,14-dihydro-15-oxo-LXA_4_ | 351 | 115 | 0.35 | ± | 0.03 | 0.23 | ± | 0.02 *** |
| 14-oxo-LXA_4_ | 349 | 115 | 0.09 | ± | 0.01 | 0.06 | ± | 0.01 |
| LTB_4_ | 335 | 195 | 1.59 | ± | 0.54 | 4.40 | ± | 1.48 |
| 5*S*,12*S*-diHETE | 335 | 195 | 0.50 | ± | 0.42 | 0.00 | ± | 0.00 |
| 6-trans-LTB_4_ | 335 | 195 | 0.39 | ± | 0.08 | 0.41 | ± | 0.07 |
| 6-trans-12-epi LTB_4_ | 335 | 195 | 0.19 | ± | 0.05 | 0.18 | ± | 0.06 |
| 20-OH-LTB_4_ | 351 | 195 | 0.01 | ± | 0.00 | 0.01 | ± | 0.00 |
| 20-COOH-LTB_4_ | 365 | 195 | 0.02 | ± | 0.00 | 0.02 | ± | 0.01 |
| PGD_2_ | 351 | 189 | 16.27 | ± | 4.44 | 23.17 | ± | 4.88 |
| PGE_2_ | 351 | 189 | 134.06 | ± | 62.40 | 129.83 | ± | 29.79 |
| PGF_2α_ | 353 | 193 | 2.18 | ± | 0.56 | 3.81 | ± | 1.29 |
| TxB_2_ | 369 | 169 | 9.86 | ± | 2.28 | 16.53 | ± | 4.67 |
